# On the Intermolecular Interactions in Thiophene-Cored Single-Stacking Junctions

**DOI:** 10.3390/ijms241713349

**Published:** 2023-08-28

**Authors:** Jiří Czernek, Jiří Brus

**Affiliations:** Institute of Macromolecular Chemistry, Czech Academy of Sciences, Heyrovsky Square 2, 16200 Prague, Czech Republic; brus@imc.cas.cz

**Keywords:** supramolecular junctions, intermolecular stacking, oligothiophenes, DFT, CCSD(T)

## Abstract

There have been attempts, both experimental and based on density-functional theory (DFT) modeling, at understanding the factors that govern the electronic conductance behavior of single-stacking junctions formed by pi-conjugated materials in nanogaps. Here, a reliable description of relevant stacked configurations of some thiophene-cored systems is provided by means of high-level quantum chemical approaches. The minimal structures of these configurations, which are found using the dispersion-corrected DFT approach, are employed in calculations that apply the coupled cluster method with singles, doubles and perturbative triples [CCSD(T)] and extrapolations to the complete basis set (CBS) limit in order to reliably quantify the strength of intermolecular binding, while their physical origin is investigated using the DFT-based symmetry-adapted perturbation theory (SAPT) of intermolecular interactions. In particular, for symmetrized S-Tn dimers (where “S” and “T” denote a thiomethyl-containing anchor group and a thiophene segment comprising “n” units, respectively), the CCSD(T)/CBS interaction energies are found to increase linearly with n ≤ 6, and significant conformational differences between the flanking 2-thiophene group in S-T1 and S-T2 are described by the CCSD(T)/CBS and SAPT/CBS computations. These results are put into the context of previous work on charge transport properties of S-Tn and other types of supramolecular junctions.

## 1. Introduction

Currently, one of the key research directions in molecular electronics [1] aims at controlling charge transport properties in supramolecular junctions [2]. The most frequently studied supramolecular junctions are formed by π-conjugated molecules (see the review [3] and also the most recent papers [4,5]). Specifically, single-stacking supramolecular junctions composed of thienyl-capped oligothiophenes were carefully investigated by Hong and his coworkers (see reference [6] and work cited therein). On the experimental side, these studies achieved reliable simultaneous measurements of conductance and the electromechanical coupling factor (denoted as α, see reference [7]) in gold electrode–single supermolecule–gold electrode junctions formed in a nanogap. This way, a series of **S**-**T***_n_* dimers (where **S** and **T** denote a thiomethyl-containing anchor group and a thiophene segment comprising *n* units, respectively; the actual structures are shown in Section 2) was examined for *n* ranging from one to four, together with control experiments for the corresponding **S**-**T***_n_*-**S** single molecules [6]. Significantly, the stacking arrangement of **S**-**T***_n_* dimers in the junctions had been previously established [8], which is a crucial structural feature of these systems that is generally not present in, for instance, the iodine-terminated oligothiophenes studied by Tao et al. [9]. On the theoretical side, density-functional theory (DFT) and molecular dynamics (MD) calculations were performed for the **S**-**T***_n_* dimers [6]. Moreover, for the related single-stacking junctions, an important spring model was derived to analyze the strain distribution in them [6], and the charge transport characteristics of some monomers and dimers positioned in between gold electrodes were simulated [6,8]. This combination of measurements and modeling provided insights into noncovalent interactions at single-molecule level and thus led to an elucidation of specific features of conductance behavior of thienyl-capped oligothiophenes, which should be important in the field of single-supermolecule electronics [10].

In this work, the aforementioned DFT computational description of **S**-**T***_n_* dimers is substantially extended. The following three main questions are addressed. (1) What are the global minima of overlapping configurations of the **S**-**T***_n_* dimers with *n* ranging from one to four? In Section 2.1, these minima are compared to the corresponding structures that were considered by Hong et al. [6]. The comparison is centered on computations of intermolecular interaction energy, ∆E, employing the domain-based local pair natural orbital (DLPNO) variant of the coupled cluster theory with singles, doubles and perturbative triples [CCSD(T)] and extrapolations to the complete basis set (CBS) limit for geometries located using the dispersion-corrected DFT approach (see Section 4 for references and technical details of the resulting DLPNO-CCSD(T)/CBS//B97-D/def2-TZVPP method). Based on this comparison, in Section 2.2 the *C_i_* symmetric geometries are chosen to investigate an extended set of pertinent structures in order to answer the second main question: (2) what is the trend in ∆E data for the **S**-**T***_n_* dimers comprising one to six thiophene units? It should be mentioned that predicted values of ∆E are vital for an interpretation of the measured probability of stacking in thiophene-based junctions [8]. The third main question, which is addressed in Section 2.3, is: (3) how big are conformational differences between the flanking 2-thiophene group in **S**-**T**_1_ and **S**-**T**_2_ dimers? These differences are crucial for an explanation of the “odd–even effect” that concerns distinct strains of intermolecular interactions in **S**-**T***_n_* dimers for odd and even values of *n*, as detailed in reference [6]. Hence, the rotation of terminal 2-thiophene rings is described in terms of the CCSD(T)/CBS ∆E-values of pertinent conformers. Furthermore, the DFT-based symmetry-adapted perturbation theory (SAPT) of intermolecular interactions [11] (see Section 4 for specifications) is applied to those conformers. Answers to questions (1)–(3) are discussed in Section 3 together with some thermodynamic considerations. They support the characterization provided by Hong et al. of the behavior of the **S**-**T***_n_* dimers in nanogaps under experimental conditions [6] and are of importance for understanding the factors that control charge transport of single-stacking junctions in general [12,13]. Moreover, the present theoretical approach can be immediately applied to other dimers in the process of modeling novel materials.

## 2. Results

### 2.1. The Energy Minima

**Figure 1:**
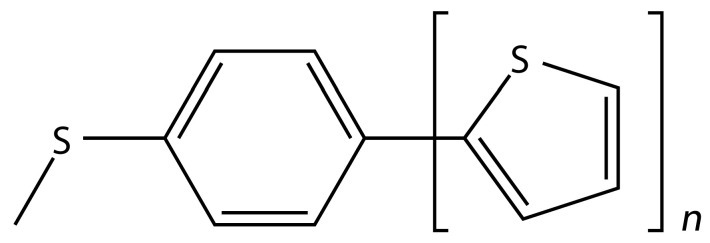
Chemical structure of the building blocks of stacked dimers described in the text.

Molecular structures of monomers forming **S**-**T***_n_* dimers are shown in Figure 1, while the optimized geometries of all dimers that are discussed in this work are provided, in xyz format, inside Supporting Information file “structures.tar”. For the **S**-**T***_n_* dimers with *n* ranging from one to four, the potential energy surface (PES) was scanned in a region relevant for the formation of stacked configurations. In short, numerous starting orientations were prepared, and minima of the PES were sought by the B97-D/def2-TZVPP approach (it should be noted that this method was recently used to search the PES of numerous dimers of heterocycles [14]). The resulting minima were ranked by the DLPNO-CCSD(T)/CBS estimation of the ∆E using the focal point procedure developed most recently [15]. As detailed in reference [15], this procedure was shown to provide ∆E-values accurate to within about 2 kJ/mol for some challenging cases from the L7 set [16] and for other complex systems. Here, it was validated by considering the conformers of the *C_i_* symmetric **S**-**T**_1_ dimer, which are described in Section 2.3. For these conformers, the canonical CCSD(T)/CBS ∆E data were obtained (see Section 4 for details) and compared to their DLPNO-CCSD(T)/CBS counterparts. Only five geometries were considered due to an exceedingly high computational cost of the underlying canonical CCSD(T) step for this **S**-**T**_1_ dimer, which contains 46 atoms. Nevertheless, the maximum and mean absolute deviations of the two data sets amount to 1.62 and 1.49 kJ/mol, respectively. The interaction energies are summarized in Supporting Information Table S1 and show only a relatively small and systematic underestimation of the DLPNO-CCSD(T)/CBS ∆E relative to the canonical CCSD(T)/CBS data (raw values of all absolute energies are provided in Supporting Information spreadsheets “DLPNO.xlsx” and “canonical.xlsx”).

Importantly, the authors of reference [6] kindly sent us coordinates of their structures of **S**-**T***_n_* dimers for *n* from one to four. An inspection of the obtained structures revealed that they were all of nearly *C_i_* symmetry. Hence, they were symmetrized and reoptimized at the B97-D/def2-TZVPP level. The resulting structures are referred to as “symmetric” from now on. Analogous *C_i_* symmetric dimers with *n* of five and six are also considered in this work (see Section 2.2). The most stable structures according to the aforementioned ranking based on the DLPNO-CCSD(T)/CBS interaction energy are denoted as “unsymmetric” in Table 1, Table 2 and Table 3 and the related discussion.

Furthermore, it was checked how structural features and interaction energies would change if some method more involved than the B97-D/def2-TZVPP is employed for the geometry optimization. In the next paragraph, such results are presented for the **S**-**T**_1_ dimer and the double-hybrid B2PLYP-D3/def2-QZVPPD approach (see Section 4 for references and technical details), which is highly accurate [17] but computationally quite demanding because it requires the second-order Møller–Plesset (MP2) correlation energy estimation using an ample def2-QZVPPD basis set (2362 basis functions in the case of the **S**-**T**_1_ dimer).

Table 1 summarizes results obtained for the B97-D/def2-TZVPP and (values in parentheses) B2PLYP-D3/def2-QZVPPD minima of two types of stacked **S**-**T**_1_ dimers. For each structure in Table 1, the total DLPNO-CCSD(T)/CBS interaction energy, which is denoted as $∆E\left(CC\right)$, is presented in terms of its three constituting parts. These parts are the Hartree–Fock ($∆E_{HF}$), MP2 correlation ($∆E_{MP2}$), and higher-order correlation ($∆E_{postMP2}$) energy contributions. Details of an estimation of the CBS-extrapolated $∆E_{HF}$, $∆E_{MP2}$, and $∆E_{postMP2}$ data are given in Section 4. Further, in Table 1, the total interaction energy obtained from the SAPT treatment, $E_{total}$, is shown together with its breakdown into the electrostatic polarization, first-order exchange, and induction and dispersion contributions (see Section 4), which are denoted as $E_{elst}$, $E_{exch}$, $E_{ind}$, and $E_{disp}$, respectively.

As immediately follows from the $∆E\left(CC\right)$ and $E_{total}$ data in Table 1, the interaction energy is only slightly affected by differences in geometry caused by the choice of the optimization method (an overlay of the B97-D/def2-TZVPP and B2PLYP-D3/def2-QZVPPD structures of the unsymmetric **S**-**T**_1_ dimer is pictured in Supporting Materials Figure S1 together with values of some intermolecular distances). It should be also noticed that the total interaction energies agree between each other to within one kJ/mol for all related structures of the **S**-**T**_1_ dimer. Moreover, ratios of the respective contributions to the total interaction energy do not significantly depend on the method that was used to optimize the geometry. This holds for both the supermolecule and SAPT calculations (see Table 1). Hence, only the B97-D/def2-TZVPP geometries were considered for larger **S**-**T***_n_* dimers, which are discussed further on in this paper.

**Figure 2:**
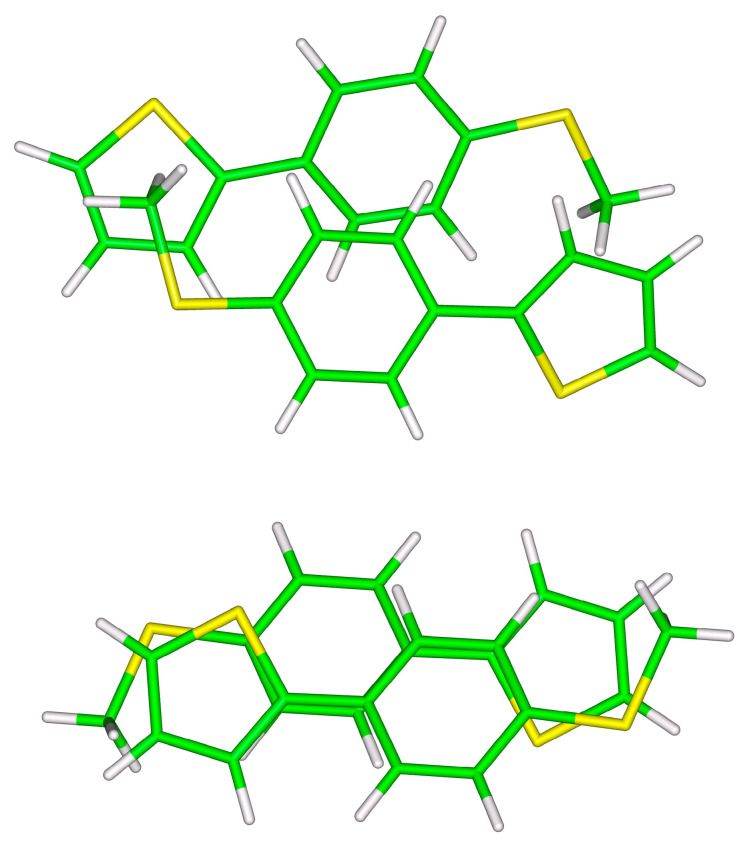
The B97-D/def2-TZVPP minima of unsymmetric (**top** panel) and symmetric (**bottom** panel) **S**-**T_1_** dimers.

The two investigated types of stacked **S**-**T**_1_ dimers in their B97-D/def2-TZVPP minima are pictured in Figure 2. Expectedly, they can be categorized as van der Waals dispersion-dominated [18]. In the unsymmetric arrangement, the dispersion-to-electrostatic ratio (see reference [19] and Section 4), $E_{disp}/E_{elst}$, is 2.25 according to the SAPT-DFT/CBS calculations carried out for the B97-D/def2-TZVPP geometry, and there are methyl groups positioned on top of 2-thiophene rings (see also Figure S1, and Tables S2 and S3). Such contacts are not present in the symmetric arrangement, and a partial overlap of 2-thiophene and phenyl rings is preferred instead in this case, with the corresponding $E_{disp}/E_{elst}$ of 2.65. Despite these structural dissimilarities, values of the total interaction energy differ only by a small amount between the two types of intermolecular complexes. In particular, the difference in $∆E\left(CC\right)$ and $E_{total}$ data amounts to 5.5 and 4.1 kJ/mol, respectively, for the B97-D/def2-TZVPP minima (see Table 1). Such small interaction energy differences indicate that there are a number of contact configurations accessible for individual supramolecular junctions at room temperature, while the presence of those configurations is of course known to affect the dispersion and shape of the α and conductance histograms [6,7,20,21].

**Figure 3:**
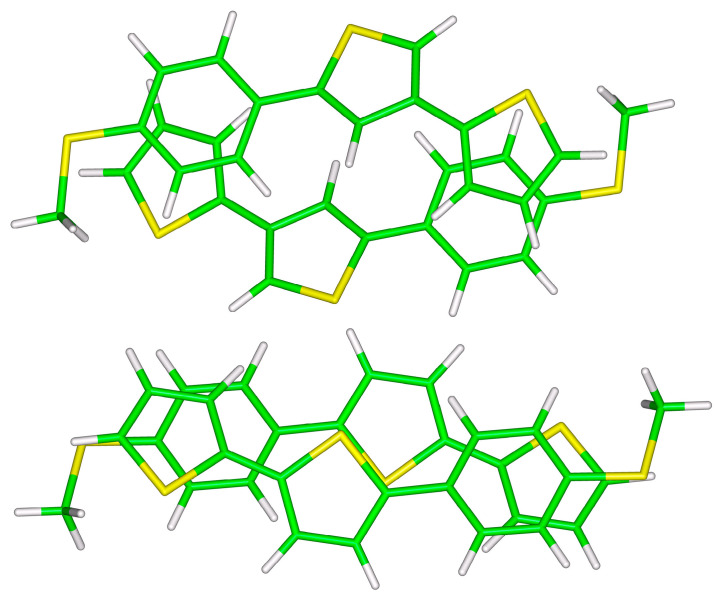
The B97-D/def2-TZVPP minima of unsymmetric (**top** panel) and symmetric (**bottom** panel) **S**-**T_2_** dimers.

As already explained in the preceding part, only the B97-D/def2-TZVPP-optimized structures of the **S**-**T**_2_ (and larger) dimers are considered here. Their interaction energies are summarized in Table 2 and demonstrate a good agreement between the $∆E\left(CC\right)$ and $E_{total}$ data. This shows that the SAPT-DFT/CBS results are fully reliable. They thus confirm that van der Waals dispersion dominates the binding between thiophene-based systems. Namely, for the unsymmetric and symmetric **S**-**T**_2_ dimer, $E_{disp}/E_{elst}$ amounts to 2.98 and 3.14, respectively (see also Table S4). Figure 3 graphically represents these structures. They feature the same intermolecular contacts as those found in the **S**-**T**_1_ dimers, that is, methyl/π(2-thiophene) and π(2-thiophene)/π(phenyl) interactions in the unsymmetric and symmetric structure, respectively. Differences in the total interaction energies between the two types of **S**-**T**_2_ dimers are also quite small (see Table 2).

**Figure 4:**
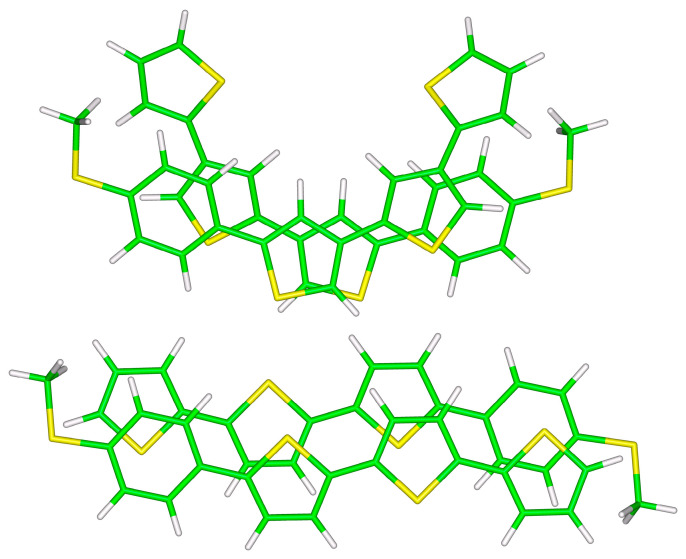
The B97-D/def2-TZVPP minima of unsymmetric (**top** panel) and symmetric (**bottom** panel) **S**-**T_3_** dimers.

**Figure 5:**
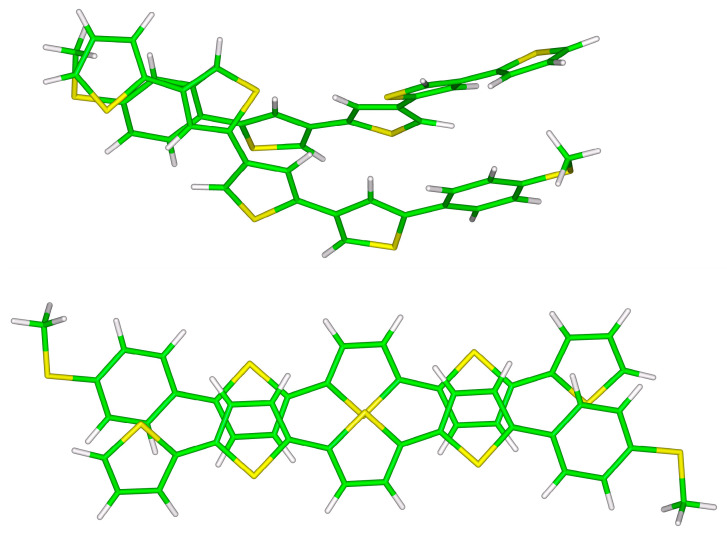
The B97-D/def2-TZVPP minima of unsymmetric (**top** panel) and symmetric (**bottom** panel) **S**-**T_4_** dimers.

In line with results described so far for the two smaller structures, differences in the total DLPNO-CCSD(T)/CBS interaction energy between the investigated configurations of **S**-**T**_3_ and **S**-**T**_4_ dimers are also relatively small (see Table 3). These larger dimers are graphically represented in Figure 4 and Figure 5. It is worth noting that the most stable unsymmetric minimum of **S**-**T**_3_ dimer does not accommodate methyl/π(2-thiophene) contacts, but instead it features π(2-thiophene)/π(phenyl) and π(central 2-thiophene)/π(central 2-thiophene) interactions. However, in the corresponding **S**-**T**_4_ dimer structure, there are methyl/π(2-thiophene) contacts present, and both its monomers have strongly bent geometries (see Figure 5). At this point, it should be mentioned that in all monomers forming symmetric dimers, the core thiophene segment is almost planar (pertinent dihedral angles have values around 20°).

### 2.2. The Molecular Size Dependence of ∆E

The DLPNO-CCSD(T)/CBS//B97-D/def2-TZVPP computational methodology was also applied to two larger dimers that had not been studied experimentally in reference [6], namely **S**-**T**_5_ and **S**-**T**_6_. Two important geometrical parameters of the minima of all structures are collected in Table 4. It should be noted that distances between thiomethyl anchor groups as expressed by the parameter $R\left(SS\right)$ are higher in the symmetric structures than in their unsymmetric counterparts for **S**-**T***_n_* dimers with *n* ranging from one to four. Importantly, the higher $R\left(SS\right)$-value is, in the case of **S**-**T**_1_, consistent with distances estimated through conductance measurements of related single-stacking junctions (see references [6,8] for details). Hence, the PES search was not performed for **S**-**T**_5_ and **S**-**T**_6_ dimers, and only their symmetric configurations are considered here. It should also be noted that for the unsymmetric minima, the separation between centers of mass of monomers (the parameter $R\left(cc\right)$ in Table 4) decreases monotonically with the increasing size of monomers. This trend is caused by a pronounced bending of monomers in bigger unsymmetric structures, which was already mentioned in the previous paragraph.

**Figure 6:**
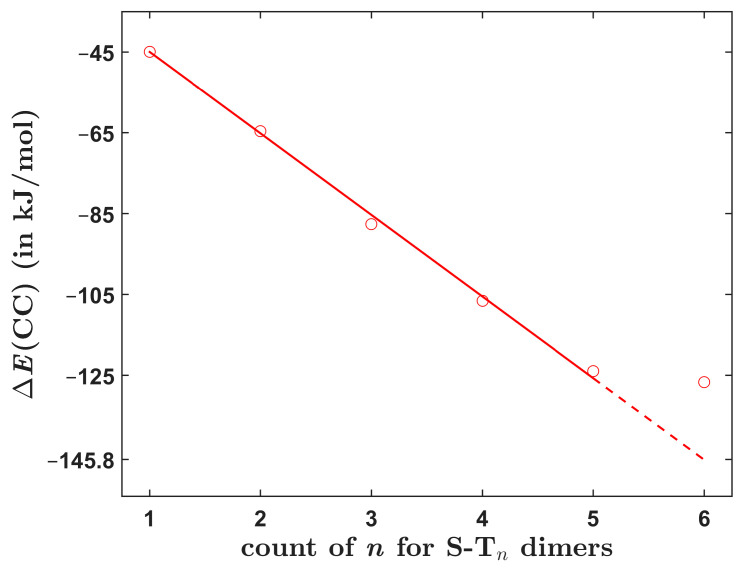
The dependence of the total interaction energy upon molecular size of investigated dimers. The red line is specified in the text.

Table 5 presents the interaction energy data for **S**-**T**_5_ and S-T_6_ dimers. An inspection of their structures reveals that they contain analogous intermolecular interactions as smaller symmetric **S**-**T***_n_* dimers, namely π/π stacking between thiophene rings in the cores and between thiophene and phenyl rings that are located closer to terminal groups. Thus, the total interaction energy might be expected to increase strictly linearly in the whole **S**-**T***_n_* series, that is, for all *n* from one to six. Interestingly, this is the case only for *n* up to and including five (see Figure 6). For these five dimers, it is convenient to fit the dependence of $∆E\left(CC\right)$ upon *n* to the linear form $\left\{∆E\left(CC\right)\right\}=a\times \left\{n-1\right\}-44.964$ kJ/mol, which uses an intercept fixed at the interaction energy value for **S**-**T**_1_. This is a robust value since it is close to the canonical CCSD(T)/CBS result of ca. −46.5 kJ/mol and also to the SAPT-DFT/CBS result of ca. −46.0 kJ/mol (see Section 2.3). The fit is highly accurate (with an average and maximum absolute residual of 1.4 and 2.3 kJ/mol, respectively, and the adjusted *R*^2^ = 0.9997). The resulting value of a slope, *a* = −20.19 with a standard error of 0.28, then approximates a monotonous increase of $∆E\left(CC\right)$ with the size of investigated dimers. Clearly, this dependence does not well describe the total interaction energy in **S**-**T**_6_, which is only 2.9 kJ/mol higher in absolute value than its counterpart for **S**-**T**_5_ (see Table 5 and Figure 6, where the dashed line is used for an extrapolation to *n* = 6). Analogous dependencies were obtained for the $∆E_{HF}$, $∆E_{MP2}$, and $∆E_{postMP2}$ data and are presented in Figures S2–S4. They show the same trend, that is, a linear growth of each ∆E term with the system size. This growth is practically uniform for the **S**-**T***_n_* dimers with *n* ≤ 5 but is much smaller for **S**-**T**_6_. The origin of the seemingly too small (in absolute value) $∆E\left(CC\right)$ of **S**-**T**_6_ is currently unknown. One might speculate about a big interatomic three-body dispersion contribution [22] that had perhaps strongly lowered the binding in **S**-**T_6_**, or even about a possible disagreement between the CCSD(T) and quantum diffusion Monte Carlo descriptions of this large (comprising 116 atoms) polarizable complex [23]. Nevertheless, it is worth noting that at the B97-D/def2-TZVPP level, the interaction energies follow a strictly linear trend for all six **S**-**T***_n_* dimers but are significantly overestimated relative to the $∆E\left(CC\right)$ data (see Figure S5 and Table S5).

### 2.3. The Conformational Dependence of ∆E

Various aspects of a relationship between conductance and conformational behavior of single-molecule junctions were described (see reference [24] and work cited therein and also the most recent references [25,26]). Less is known about this relationship in dimer junctions [27]. Rotations of terminal 2-thiophene rings were considered in an analysis of conductance and α-value measurements by Hong and coworkers [6]. Specifically, DFT and MD calculations were used to interpret the experimentally observed “odd-even” effect in α-values for **S**-**T***_n_* dimers with *n* ranging from one to four [6]. Here, the dependence of binding strength upon local conformational changes of flanking 2-thiophenes was investigated by means of the CCSD(T)/CBS and DFT-SAPT/CBS computations. The symmetrized B97-D/def2-TZVPP geometries of **S**-**T**_1_ and **S**-**T**_2_ dimers were employed to vary the dihedral angles that are denoted as *β* in the following and express a departure of the terminal 2-thiophene ring from planarity relative to the preceding ring. For these dimers, Figures S6 and S7 show chains of atoms defining the respective *β* angle. Due to the *C_i_* symmetry of the dimers, their constituting monomers have a dihedral angle with a value of either +*β* or −*β*. It is noted that analogous dihedral angles were reported as (180° − *β*) in reference [6].

**Figure 7:**
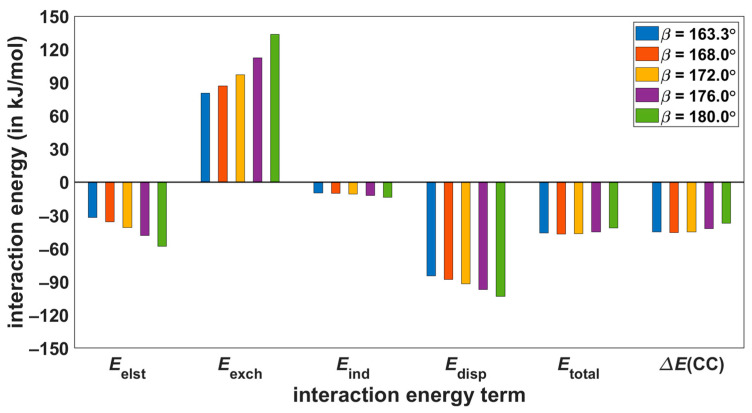
Values extrapolated to the complete basis set limit of the intermolecular interaction energy terms for **S**-**T**_1_ structures with varying dihedral angle *β*.

**Figure 8:**
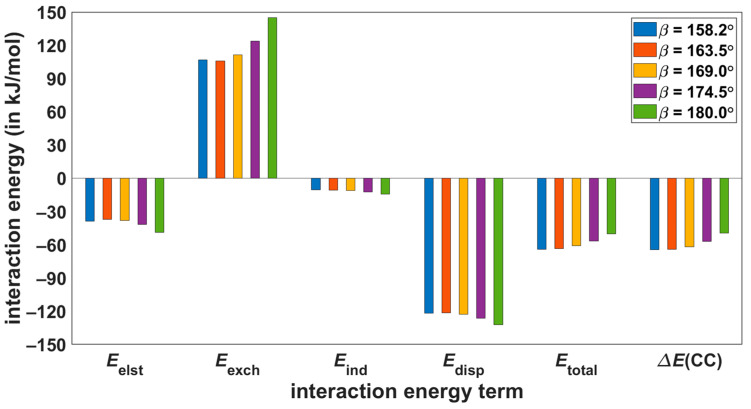
Values extrapolated to the complete basis set limit of the intermolecular interaction energy terms for **S**-**T**_2_ structures with varying dihedral angle *β*.

The dependence of individual interaction energies upon the angle *β* is graphically represented in Figure 7 and Figure 8 for **S**-**T**_1_ and **S**-**T**_2_ systems, respectively (additional details can be found in Tables S1, S6 and S7). For both dimers, structures with the lowest *β*-value correspond to the pertinent B97-D/def2-TZVPP minimum, and the conformational region between this value and *β* = 180° is sampled. Importantly, the high-level calculations show that a decrease in the absolute value of the total interaction energy with increasing value of *β* is about two times lower in **S**-**T**_1_ than in **S**-**T**_2_ dimers. For example, in the **S**-**T**_1_ dimer, the $∆E\left(CC\right)$ changes by |7.7| kJ/mol between structures with *β* of 163.3° and 180.0°, while in the **S**-**T**_2_ dimer, the $∆E\left(CC\right)$ changes by |14.7| kJ/mol between structures with *β* = 163.5° and 180.0°. Such variations of course indicate a higher degree of local conformation freedom in **S**-**T**_1_ as compared to the **S**-**T**_2_ dimer, in agreement with reference [6]. This trend in the $∆E\left(CC\right)$ data is confirmed by DFT-SAPT/CBS results. In particular, for the same example as above, the differences in $E_{total}$ are |4.6| and |13.1| kJ/mol for the corresponding *β* changes in the **S**-**T**_1_ and **S**-**T**_2_ dimers, respectively. It should also be mentioned that the CCSD(T) and DFT-SAPT methods place a minimum of the total interaction energy of **S**-**T**_1_ between the first and third (in ascending order) *β*-value (see Figure 7 and Table S1), while these methods agree that absolute values of the total interaction energy decrease monotonically with increasing *β* in the case of **S**-**T**_2_ dimers (see Figure 8 and Table S2). Thus, the DFT-SAPT/CBS data should be of interest as they are quite accurate and describe the physical origin of intermolecular interactions. Analysis of these data reveals that for all investigated *β* angles, the intermolecular binding in both the **S**-**T**_1_ and **S**-**T**_2_ dimers is dominated by van der Waals dispersion (underlying values of the interaction energy components are collected in Tables S6 and S7). Specifically, in the **S**-**T**_1_ system, the $E_{disp}/E_{elst}$ ratio amounts to 2.65 for *β* = 163.3°, and for *β* = 180.0° it is reduced to 1.79. This ratio equals 3.14 for *β* = 158.2° and drops to 2.71 for *β* = 180.0° in the **S**-**T**_2_ dimers.

## 3. Discussion

Here, various dimers of thiophene-cored structures were computationally studied in their stacked arrangements with the general goal of accurately describing intermolecular interactions in this type of complex. Importantly, in reference [6], stacked configurations of the four smaller oligothiophene systems were characterized by the mechanical and conductance measurements of dimer junctions; an “odd-even” trend in α-values was found. This trend, which represents differences in the strain distribution of intermolecular interactions of single-stacking junctions, was then interpreted using DFT and MD modeling [6]. Results obtained in the present work provide an extensive description of structures and intermolecular binding of related **S**-**T***_n_* dimers with *n* ranging from one to six. Several specific questions about these adducts, which were posed in the Introduction, are discussed in this section.

The PES search was performed at the DLPNO-CCSD(T)/CBS//B97-D/def2-TZVPP level for the **S**-**T***_n_* complexes with *n* ≤ 4 in their overlapping configurations. It unveiled the minima that were more strongly bound than their counterparts of the *C_i_* molecular symmetry. Interestingly, all these enthalpically more favorable structures feature significantly shorter distances between thiomethyl anchors than the corresponding *C_i_* symmetric geometries do. However, it appeared that geometries with longer $R\left(SS\right)$-values would be more representative of the junctions investigated experimentally [6,8], and only the symmetric geometries were considered while following structure dependencies that are described below.

**Figure 9:**
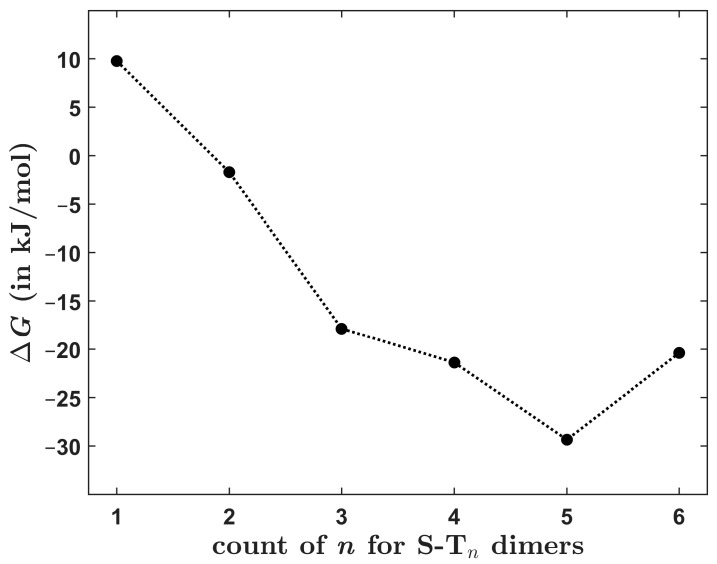
The theoretically estimated Gibbs free energy change at 298.15 K of the formation of investigated dimers plotted against their size. Data points are connected by the dotted line.

The dependence of the interaction energy upon the molecular size was inspected for the six symmetric dimers. Absolute values of $∆E\left(CC\right)$ were found to monotonously increase with the system size. This rise was fairly large and almost uniform (20.19 ± 0.28 kJ/mol according to the linear model specified in Section 2.2) for the **S**-**T***_n_* dimers with *n* ≤ 5 but was only negligible (amounting to 2.9 kJ/mol) for the biggest system, that is, **S**-**T**_6_. Hence, it may be worthwhile to also predict a change in the Gibbs free energy accompanying the formation of dimers under standard thermodynamic conditions, ∆G. Only a crude method for the ∆G estimation was used, which employed the DLPNO-CCSD(T)/CBS interaction energies together with results of the B97-D/def2-TZVPP harmonic vibrational analysis of each dimer and its constituting monomers. Then, for the assumed dimerization process at the given temperature, related changes in the zero-point vibrational energies, the vibrational thermal energies, and the entropies were calculated routinely [28] and were combined with the $∆E\left(CC\right)$ data to arrive at a value for the Gibbs free energy of formation. Results obtained for a temperature of 298.15 K are graphically represented in Figure 9 (underlying values of the thermodynamic parameters are collected in Table S8). They show a complicated behavior of ∆G as a function of molecular size. Namely, a positive ∆G-value is predicted for **S**-**T**_1_, in contrast to results obtained for the rest of the investigated dimers. Furthermore, the ∆G of **S**-**T**_4_ is slightly higher in absolute value than its counterpart for **S**-**T**_6_. This demonstrates that there can be “sweet spots” of thermodynamic stabilization of stacked dimers for certain sizes of constituting monomers. In the present case, such a “sweet spot” was apparently found at *n* = 5 (see Figure 9).

Moreover, dependencies of the interaction energy upon varying dihedral angles, which define orientations of terminal 2-thiophene rings with respect to the core of **S**-**T**_1_ and **S**-**T**_2_ dimers, were followed. It was suggested in reference [6] that differences in rotational barriers of these rings in single-stacking oligothiophene junctions led to the “odd-even” effect in the measured α-values. In this work, the rings were rotated between their position in the B97-D/def2-TZVPP minimum and their planarity relative to the rest of the dimer. For the rotated structures, the CCSD(T)/CBS and SAPT-DFT/CBS interaction energies were obtained. These calculations revealed much lower changes in the interaction energy with the rotation in **S**-**T**_1_ as compared to the **S**-**T**_2_ dimer. This result is consistent with a higher conformational freedom in the smaller system and is thus in line with an interpretation of the “odd-even” effect provided in reference [6].

## 4. Materials and Methods

Geometry optimizations and subsequent estimations of harmonic vibrational frequencies and intermolecular interaction energies were performed using the Gaussian 16, revision C.01 suite of codes [29] with default settings. These computations used the B97-D/def2-TZVPP (the B97 functional [30] combined with the empirical dispersion correction from reference [31] and applied together with the TZVPP basis set [32]) and the B2PLYP-D3/def2-QZVPD (the double-hybrid B2-PLYP functional [33,34] combined with the D3 empirical dispersion correction [35] and applied together with the QZVPPD basis set [36]; see also reference [37]) approaches.

The canonical CCSD(T)/CBS interaction energies were obtained by the focal-point method expressed by Equation (1) (see reference [15] for further details):

(1) $${\Delta E}_{CCSD\left(T\right)}^{CBS}={∆E}_{HF}^{a5Z}+{∆E}_{MP2}^{a5Z}+{∆E}_{post-MP2}^{aTZ},$$

where subscripts denote the respective energy terms, namely the total Hartree–Fock energy (HF), the MP2 correlation energy (MP2), and the higher-order correlation energy (post-MP2), and superscripts specify the basis set used to compute the respective term. Abbreviations “aTZ”, “aQZ”, and “a5Z” denote the standard augmented correlation-consistent polarized valence triple-zeta, quadruple-zeta, and quintuple-zeta basis sets, respectively [38,39]. The MP2/a5Z correlation energies were obtained in the resolution-of-the-identity integral approximation [40,41] while using the relevant auxiliary basis sets [41]. Calculations of the HF/a5Z and MP2/a5Z energies were performed in Turbomole, version 7.1 [42]. Calculations of the canonical CCSD(T)/aTZ and MP2/aTZ correlation energies were carried out in Molpro 2021.2 [43].

The SAPT-DFT/CBS interaction energies were estimated using the same procedures as in our most recent work [44]. The $E_{elst}$, $E_{exch}$, $E_{disp}$, and $E_{ind}$ contributions to the total interaction energy, $E_{total}$, from Section 2 are related to the underlying interaction energy terms as follows: $E_{elst}$ and $E_{exch}$ are the polarization and exchange energy contributions, respectively, arising in the first order of the perturbation theory of intermolecular interactions [45]; $E_{disp}$ is the dispersion energy contribution obtained as a sum of the second order terms $E_{disp.}^{SAPT\;(2)}$ and $E_{disp.-exch.}^{SAPT\;(2)}$ [46]; and $E_{ind}$ is the induction energy contribution approximated by a sum of the second order terms $E_{ind.}^{SAPT\;(2)}$ and $E_{ind.}^{SAPT\;(2)}$ [47] and the correction term $E_{\delta (HF)}^{SAPT\;}$, which is computed at the HF level [48]. All the related calculations were performed in Molpro 2021.2.

The DLPNO-CCSD(T)/CBS interaction energies were approximated by the focal-point method from reference [15], which applies Equation (2) (the notation is as in Equation (1), and a right arrow is used to indicate an application of the two-point extrapolation formula from reference [49]):

(2) $${\Delta E}_{DLPNO-CCSD\left(T\right)}^{CBS}={∆E}_{HF}^{aQZ}+{∆E}_{MP2}^{aTZ\to aQZ}+{∆E}_{post-MP2}^{aTZ\to aQZ},$$

while the CCSD(T) and MP2 correlation energies were obtained in the DLPNO approximation [50,51,52,53]. The ORCA 5.0.3 program package [54] was used with the “TightPNO” set of parameters for the truncation of the electron-correlation space and with the default method of orbital localization.

## 5. Conclusions

The main findings of this work can be summarized according to the three key questions that were posed in the Introduction, as follows. Firstly, ∆E-values obtained for global minima of overlapping configurations of the **S**-**T***_n_* dimers with *n* ranging from one to four are only slightly higher than for the corresponding *C_i_* symmetric structures. Secondly, ∆E-values grow linearly with the system size for the **S**-**T***_n_* dimers with *n* up to and including six. Thirdly, in agreement with an interpretation of the “odd-even” effect that was found experimentally in the junctions [6], a hindrance of the rotation of terminal 2-thiophene rings is much higher in **S**-**T**_2_ than in **S**-**T**_1_. In addition, the presented DFT-SAPT analysis of intermolecular interactions highlights the dominant role of van der Waals dispersion in the stabilization of thiophene-cored dimers. These results are expected to be of interest in the computational design of novel materials.

## Figures and Tables

**Table 1:** The intermolecular interaction energy components as obtained by computational procedures specified in the text for the **S**-**T_1_** dimers. All values are in kJ/mol.

Configuration	Supermolecular				SAPT-DFT/CBS				
	$∆E_{HF}$	$∆E_{MP2}$	$∆E_{postMP2}$	$∆E\left(CC\right)$	$E_{elst}$	$E_{exch}$	$E_{ind}$	$E_{disp}$	$E_{total}$
unsymmetric	29.7 (25.5)	−105.2 (−99.4)	25.1 (23.6)	−50.5 (−50.4)	−38.9 (−33.0)	87.1 (74.7)	−10.9 (−9.5)	−87.5 (−82.5)	−50.1 (−50.2)
symmetric	35.3 (33.5)	−107.5 (−105.9)	27.2 (26.7)	−45.0 (−45.7)	−32.1 (−31.2)	80.6 (77.6)	−9.7 (−9.0)	−84.8 (−83.8)	−46.0 (−46.3)

**Table 2:** The intermolecular interaction energy components as obtained by computational procedures specified in the text for the **S**-**T_2_** dimers. All values are in kJ/mol.

Configuration	Supramolecular				SAPT-DFT/CBS				
	$∆E_{HF}$	$∆E_{MP2}$	$∆E_{postMP2}$	$∆E\left(CC\right)$	$E_{elst}$	$E_{exch}$	$E_{ind}$	$E_{disp}$	$E_{total}$
unsymmetric	51.6	−160.7	41.9	−67.2	−41.9	113.6	−12.7	−124.9	−65.9
symmetric	52.3	−159.7	42.7	−64.6	−38.8	106.9	−10.5	−121.8	−64.2

**Table 3:** The intermolecular interaction energy components as obtained by computational procedures specified in the text for the **S**-**T_3_** and (in parentheses) **S**-**T_4_** dimers. All values are in kJ/mol.

Configuration	$∆E_{HF}$	$∆E_{MP2}$	$∆E_{postMP2}$	$∆E\left(CC\right)$
unsymmetric	71.1 (74.8)	−224.8 (−259.6)	61.0 (68.5)	−92.8 (−116.3)
symmetric	68.3 (81.3)	−215.0 (−261.6)	59.2 (74.6)	−87.6 (−105.8)

**Table 4:** Selected distances in the symmetric and (in parentheses) unsymmetric dimers; (R(cc) and R(SS) denote separations between centroids and sulfur atoms of thiomethyl groups of constituting monomers, respectively. All values are in pm.

System	R(cc)	R(SS)
**S**-**T**_1_	395 (399)	1025 (834)
**S**-**T**_2_	380 (359)	1396 (1225)
**S**-**T**_3_	391 (297)	1784 (1538)
**S**-**T**_4_	400 (241)	2189 (1724)
**S**-**T**_5_	402	2260
**S**-**T**_6_	445	2585

**Table 5:** The intermolecular interaction energy components as obtained by computational procedures specified in the text for **S**-**T_5_** and **S**-**T_6_** dimers. All values are in kJ/mol.

System (in *C_i_* Symmetry)	$∆E_{HF}$	$∆E_{MP2}$	$∆E_{postMP2}$	∆E(CC)
**S-T_5_**	101.2	−314.1	89.0	−123.9
**S-T_6_**	108.1	−329.3	94.4	−126.7

## Data Availability

The data presented in this study are available in the article and in the Supplementary Materials.

## Supplementary Materials

The supporting information can be downloaded at: https://www.mdpi.com/article/10.3390/ijms241713349/s1.
