# Supplementary material for: On the Intermolecular Interactions in Thiophene-Cored Single-Stacking Junctions
Source: Int J Mol Sci. 2023 Aug 28;24(17):13349. doi: 10.3390/ijms241713349 (PMC10487960; doi:10.3390/ijms241713349)

Supplementary Information to “On the Intermolecular Interactions in Thiophene-Cored Single-Stacking Junctions” by Czernek & Brus (*IJMS* 2023)

Table of Contents

Table S1 – the canonical and DLPNO CCSD(T)/CBS results … page S2

Table S2 – details of the SAPT-DFT/CBS results for the **S-T**$_1$ dimers in their B97-D geometry … page S3

Table S3 – details of the SAPT-DFT/CBS results for the **S-T**$_1$ dimers in their B2PLYP-D3 geometry … page S4

Table S4 – details of the SAPT-DFT/CBS results for the **S-T**$_2$ dimers in their B97-D geometry … page S5

Table S5 – the B97-D and DLPNO CCSD(T)/CBS interaction energies … page S5

Table S6 – underlying data for Figure 7 … page S6

Table S7 – underlying data for Figure 8 … page S7

Table S8 – underlying data for Figure 9 … page S8

Figure S1 – structures of the unsymmetric **S-T**$_1$ dimer … page S8

Figure S2 – dependence of the ΔE_{HF} data upon molecular size … page S9

Figure S3 – dependence of the ΔE_{MP2} data upon molecular size … page S10

Figure S4 – dependence of the $\Delta E_{\mathrm{post\,MP2}}$ data upon molecular size … page S11

Figure S5 – dependence of the B97-D interaction energy upon molecular size … page S12

Figure S6 – definition of the dihedral angle β in **S-T**$_1$ dimers … page S13

Figure S7 – definition of the dihedral angle β in **S-T**$_2$ dimers … page S13

Table S1. The variation of the interaction energy terms (in J/mol) of the **S-T**1 dimers with the dihedral angle β (in degrees), which is defined in Figure S5. The respective terms were obtained using procedures expressed by Eq. 1 and (for values in parentheses) Eq. 2, and $\Delta E = \Delta E_{\mathrm{HF}} + \Delta E_{\mathrm{MP2}} + \Delta E_{\mathrm{post\,MP2}}$.

β	ΔE_{HF}	ΔE_{MP2}	$\Delta E_{\mathrm{post\,MP2}}$	ΔE
163.3	35324<br>(35345)	–106415<br>(–107485)	24635<br>(27176)	–46455<br>(–44964)
168.0	37611<br>(37639)	– 110698<br>(–111975)	25908<br>(28669)	–47180<br>(–45668)
172.0	42207<br>(42245)	–11573<br>(–117595)	27250<br>(30364)	–46297<br>(–44985)
176.0	49694<br>(49749)	–122176<br>(–124049)	28878<br>(32239)	–43604<br>(–42061)
180.0	60679<br>(60756)	–130380<br>(–132178)	30834<br>(34171)	–38868<br>(–37252)

Table S2. Raw data, in J/mol, used to obtain the SAPT-DFT/CBS interaction energies of **S-T**1 dimers in their B97-D/def2-TZVPP geometry that are presented in Table 1, where the respective terms are: $E_{\mathrm{elst}} = E^{\mathrm{SAPT\,(1)}}_{\mathrm{elst.}}$; $E_{\mathrm{exch}} = E^{\mathrm{SAPT\,(1)}}_{\mathrm{exch.}}$; $E_{\mathrm{ind}} = E^{\mathrm{SAPT\,(2)}}_{\mathrm{ind.}} + E^{\mathrm{SAPT\,(2)}}_{\mathrm{ind.-exc}}$. $+ E^{\mathrm{SAPT}}_{\delta(\mathrm{HF})}$; $E_{\mathrm{disp}} = E^{\mathrm{SAPT\,(2)}}_{\mathrm{disp.}} + E^{\mathrm{SAPT\,(2)}}_{\mathrm{disp.-exch.}}$; and $E_{\mathrm{total}} = E_{\mathrm{elst}} + E_{\mathrm{exch}} + E_{\mathrm{ind}} + E_{\mathrm{disp}}$.

interaction energy term	the unsymmetric minimum	the symmetric minimum
$E^{\mathrm{SAPT\,(1)}}_{\mathrm{elst.}}$	–38873	–32073
$E^{\mathrm{SAPT\,(1)}}_{\mathrm{exch.}}$	87127	80591
$E^{\mathrm{SAPT\,(2)}}_{\mathrm{disp.}}$	–42376	–40805
$E^{\mathrm{SAPT\,(2)}}_{\mathrm{disp.-exch.}}$	38840	37799
$E^{\mathrm{SAPT\,(2)}}_{\mathrm{ind.}}$	–103060	–99805
$E^{\mathrm{SAPT\,(2)}}_{\mathrm{ind.-exch.}}$	15584	14962
$E^{\mathrm{SAPT}}_{\delta(\mathrm{HF})}$	–7360	–6670
E_{total}	–50118	–46002

Table S3. Raw data, in J/mol, used to obtain the SAPT-DFT/CBS interaction energies of **S-T**$_1$ dimers in their B2PLYP-D3/def2-QZVPPD geometry that are presented in Table 1, where the respective terms are: $E_{\mathrm{elst}} = E_{\mathrm{elst.}}^{\mathrm{SAPT\,(1)}}$; $E_{\mathrm{exch}} = E_{\mathrm{exch.}}^{\mathrm{SAPT\,(1)}}$; $E_{\mathrm{ind}} = E_{\mathrm{ind.}}^{\mathrm{SAPT\,(2)}} + E_{\mathrm{ind.-exch.}}^{\mathrm{SAPT\,(2)}} + E_{\delta(\mathrm{HF})}^{\mathrm{SAPT}}$; $E_{\mathrm{disp}} = E_{\mathrm{disp.}}^{\mathrm{SAPT\,(2)}} + E_{\mathrm{disp.-exc}}^{\mathrm{SAPT\,(2)}}$; and $E_{\mathrm{total}} = E_{\mathrm{elst}} + E_{\mathrm{exch}} + E_{\mathrm{ind}} + E_{\mathrm{disp}}$.

interaction energy term	the unsymmetric minimum	the symmetric minimum
$E_{\mathrm{elst.}}^{\mathrm{SAPT\,(1)}}$	–32951	–31160
$E_{\mathrm{exch.}}^{\mathrm{SAPT\,(1)}}$	74690	77551
$E_{\mathrm{disp.}}^{\mathrm{SAPT\,(2)}}$	–35266	–40096
$E_{\mathrm{disp.-exch.}}^{\mathrm{SAPT\,(2)}}$	32031	37300
$E_{\mathrm{ind.}}^{\mathrm{SAPT\,(2)}}$	–96251	–98367
$E_{\mathrm{ind.-exch.}}^{\mathrm{SAPT\,(2)}}$	13754	14615
$E_{\delta(\mathrm{HF})}^{\mathrm{SAPT}}$	–6253	–6185
E_{total}	–50246	–46343

Table S4. Raw data, in J/mol, used to obtain the SAPT-DFT/CBS interaction energies of **S-T**$_2$ dimers in their B97-D/def2-TZVPP geometry that are presented in Table 2, where the respective terms are: $E_{\text{elst}} = E^{\text{SAPT (1)}}_{\text{elst.}}$; $E_{\text{exch}} = E^{\text{SAPT (1)}}_{\text{exch.}}$; $E_{\text{ind}} = E^{\text{SAPT (2)}}_{\text{ind.}} + E^{\text{SAPT (2)}}_{\text{ind.−exch.}} + E^{\text{SAPT}}_{\delta\text{(HF)}}$; $E_{\text{disp}} = E^{\text{SAPT (2)}}_{\text{disp.}} + E^{\text{SAPT (2)}}_{\text{disp.−exc}}$.; and $E_{\text{total}} = E_{\text{elst}} + E_{\text{exch}} + E_{\text{ind}} + E_{\text{disp}}$.

interaction energy term	the unsymmetric minimum	the symmetric minimum
$E^{\text{SAPT (1)}}_{\text{elst.}}$	–41851	–38830
$E^{\text{SAPT (1)}}_{\text{exch.}}$	113561	106909
$E^{\text{SAPT (2)}}_{\text{disp.}}$	–56534	–56160
$E^{\text{SAPT (2)}}_{\text{disp.−exch.}}$	52904	53269
$E^{\text{SAPT (2)}}_{\text{ind.}}$	–146632	–142805
$E^{\text{SAPT (2)}}_{\text{ind.−exch.}}$	21684	21002
$E^{\text{SAPT}}_{\delta\text{(HF)}}$	–9064	–7630
E_{total}	–65932	–64245

Table S5. The B97-D/def2-TZVPP interaction energy (denoted as $\Delta E(\text{DFT})$) and its $\Delta E(\text{CC})$ counterpart for the symmetric **S-T**$_n$ dimers. All values are in kJ/mol.

system	$\Delta E(\text{DFT})$	$\Delta E(\text{CC})$
**S-T**$_1$	–40961	–44964
**S-T**$_2$	–56651	–64634
**S-T**$_3$	–75354	–87590
**S-T**$_4$	–88826	–105801
**S-T**$_5$	–101839	–123937
**S-T**$_6$	–125562	–126727

Table S6. Raw data, in J/mol, used to obtain the SAPT-DFT/CBS interaction energies of **S-T**1 dimers that are presented in Figure 7, where the respective terms are: $E_{\mathrm{elst}} = E^{\mathrm{SAPT\,(1)}}_{\mathrm{elst.}}$; $E_{\mathrm{exch}} = E^{\mathrm{SAPT\,(1)}}_{\mathrm{exch.}}$; $E_{\mathrm{ind}} = E^{\mathrm{SAPT\,(2)}}_{\mathrm{ind.}} + E^{\mathrm{SAPT\,(2)}}_{\mathrm{ind.-exc}} + E^{\mathrm{SAPT}}_{\delta(\mathrm{HF})}$; $E_{\mathrm{disp}} = E^{\mathrm{SAPT\,(2)}}_{\mathrm{disp.}} + E^{\mathrm{SAPT\,(2)}}_{\mathrm{disp.-exch.}}$; and $E_{\mathrm{total}} = E_{\mathrm{elst}} + E_{\mathrm{exch}} + E_{\mathrm{ind}} + E_{\mathrm{disp}}$. For each structure, also the $\Delta E(\mathrm{CC})$ value is shown (in J/mol).

term	the dihedral angle β (in degrees)				
	163.2	168.0	172.0	176.0	180.0
$E^{\mathrm{SAPT\,(1)}}_{\mathrm{elst.}}$	–32073	–35884	–40947	–48104	–57887
$E^{\mathrm{SAPT\,(1)}}_{\mathrm{exch.}}$	80591	87020	97076	112230	133641
$E^{\mathrm{SAPT\,(2)}}_{\mathrm{disp.}}$	–40805	–46655	–54962	–67274	–84834
$E^{\mathrm{SAPT\,(2)}}_{\mathrm{disp.-exch.}}$	37799	43581	51750	63780	80784
$E^{\mathrm{SAPT\,(2)}}_{\mathrm{ind.}}$	–99805	–104180	–109644	–116850	–125953
$E^{\mathrm{SAPT\,(2)}}_{\mathrm{ind.-exch.}}$	14962	16135	17695	19830	22599
$E^{\mathrm{SAPT}}_{\delta(\mathrm{HF})}$	–6670	–7025	–7628	–8527	–9711
E_{total}	–46002	–47007	–46659	–44915	–41361
$\Delta E(\mathrm{CC})$	–44964	–45668	–44985	–42061	–37252

Table S7. Raw data, in J/mol, used to obtain the SAPT-DFT/CBS interaction energies of **S-T**$_2$ dimers that are presented in Figure 8, where the respective terms are: $E_{\text{elst}} = E^{\text{SAPT (1)}}_{\text{elst.}}$; $E_{\text{exch}} = E^{\text{SAPT (1)}}_{\text{exch.}}$; $E_{\text{ind}} = E^{\text{SAPT (2)}}_{\text{ind.}} + E^{\text{SAPT (2)}}_{\text{ind.-exch.}} + E^{\text{SAPT}}_{\delta\text{(HF)}}$; $E_{\text{disp}} = E^{\text{SAPT (2)}}_{\text{disp.}} + E^{\text{SAPT (2)}}_{\text{disp.-exch.}}$; and $E_{\text{total}} = E_{\text{elst}} + E_{\text{exch}} + E_{\text{ind}} + E_{\text{disp}}$. For each structure, also the $\Delta E(\text{CC})$ value is shown (in J/mol).

term	the dihedral angle β (in degrees)				
	158.2	163.5	169.0	174.5	180.0
$E^{\text{SAPT (1)}}_{\text{elst.}}$	–38830	–37292	–38118	–41719	–48765
$E^{\text{SAPT (1)}}_{\text{exch.}}$	106909	105927	111241	123787	145096
$E^{\text{SAPT (2)}}_{\text{disp.}}$	–56160	–55865	–59526	–68207	–83621
$E^{\text{SAPT (2)}}_{\text{disp.-exch.}}$	53269	52840	56265	64569	79336
$E^{\text{SAPT (2)}}_{\text{ind.}}$	–142805	–142060	–144026	–148952	–157053
$E^{\text{SAPT (2)}}_{\text{ind.-exch.}}$	21002	20683	21144	22487	24814
$E^{\text{SAPT}}_{\delta\text{(HF)}}$	–7630	–7603	–7962	–8761	–10074
E_{total}	–64245	–63368	–60982	–56795	–50265
$\Delta E(\text{CC})$	–64634	–64189	–61754	–56953	–49561

Table S8. The energetics data at T = 298.15 K related to the formation of **S-T**$_n$ dimers. All values are in J/mol.

system	$\Delta E(\mathrm{CC})$	$\Delta E(\mathrm{VZPE})$	ΔH_T	$-T\Delta S$	ΔG
**S-T**$_1$	–44964	3998	2402	48341	9778
**S-T**$_2$	–64634	4298	2013	56606	–1717
**S-T**$_3$	–87590	4431	1662	63631	–17866
**S-T**$_4$	–105801	5370	973	78083	–21375
**S-T**$_5$	–123937	5316	558	88714	–29350
**S-T**$_6$	–126727	7736	–608	99217	–20382

Figure S1. The overlay of optimized structures of the unsymmetric **S-T**$_1$ dimer: the B97-D/def2-TZVPP and B2PLYP-D3/def2-QZVPPD minimum is colored in magenta and cyan, respectively. Distances between the methyl carbon and the centroid of 2-thiophene ring are shown (schematically indicated by the black line).

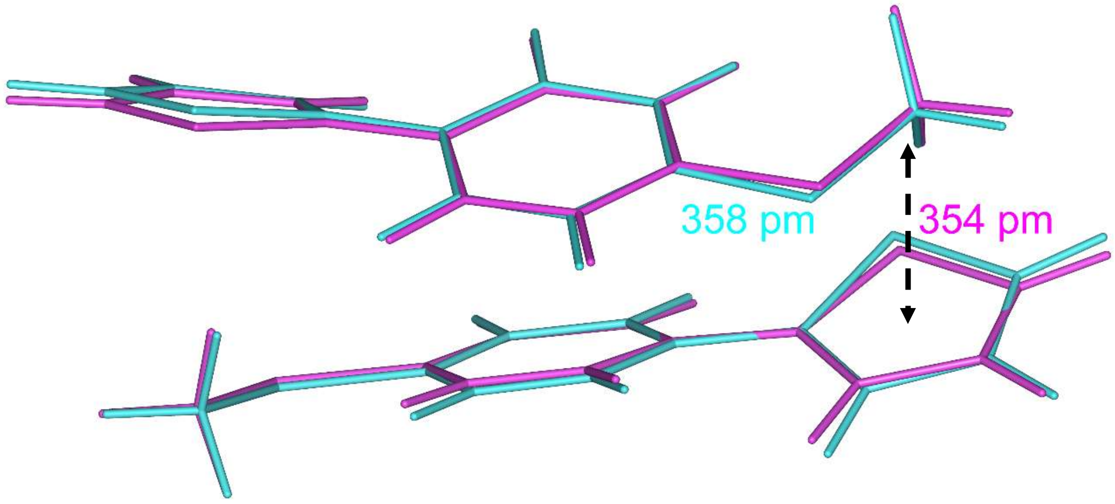

Figure S2. The dependence of the HF portion of the total interaction energy upon molecular size of investigated dimers. The red line is $\{\Delta E(\mathrm{HF})\} = a \times \{n-1\} + 35.345$ kJ/mol with $a = 16.13$ that has the standard deviation is 0.27.

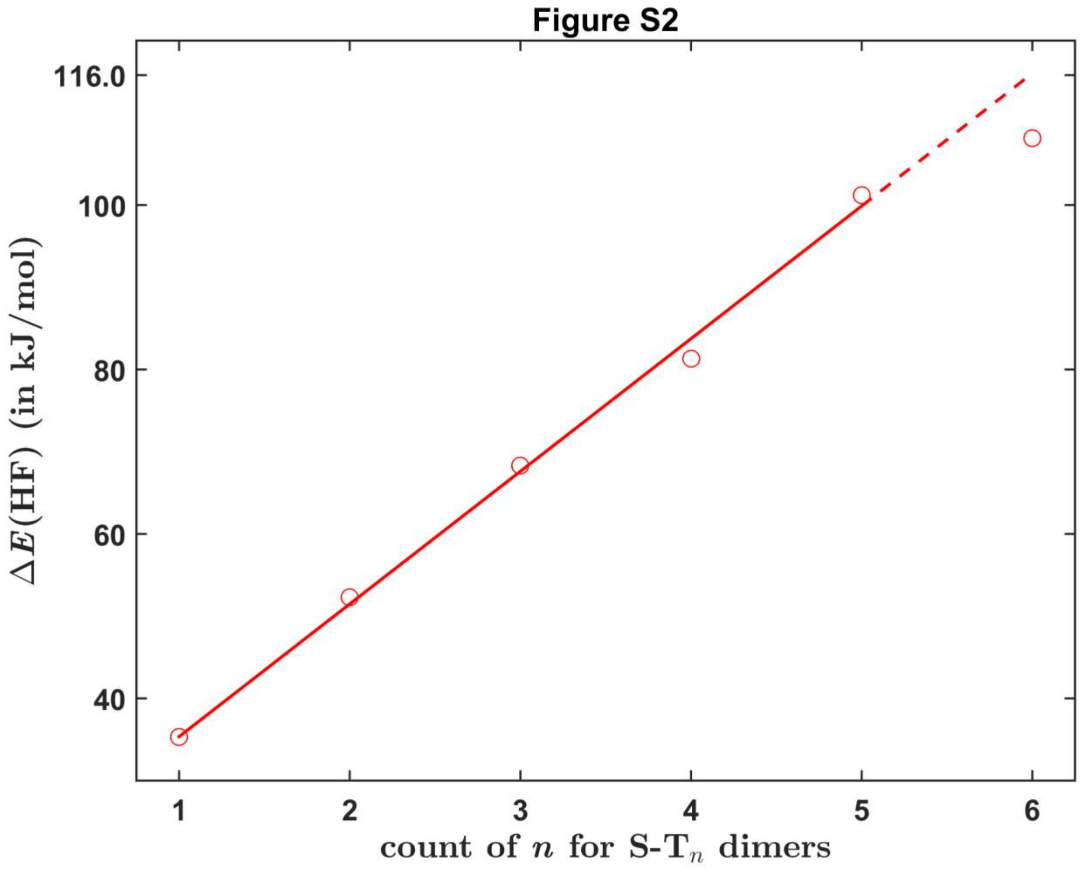

Figure S3. The dependence of the MP2 portion of the total interaction energy upon molecular size of investigated dimers. The red line is $\{\Delta E(\mathrm{MP2})\} = a \times \{n-1\} - 107.485$ kJ/mol with $a = -51.88$ that has the standard deviation is 0.38.

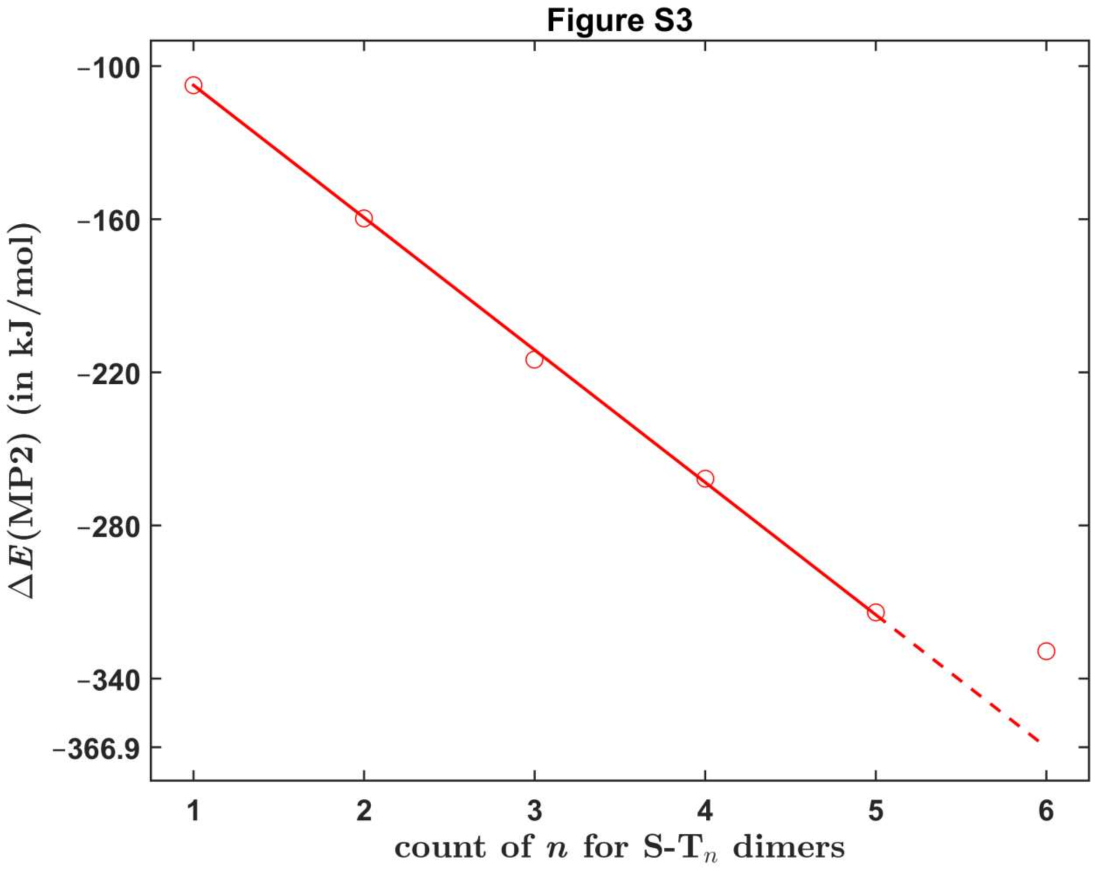

Figure S4. The dependence of the post MP2 portion of the total interaction energy upon molecular size of investigated dimers. The red line is $\{\Delta E(\text{post MP2})\} = a \times \{n-1\} + 27.176$ kJ/mol with $a = 15.64$ that has the standard deviation of 0.10.

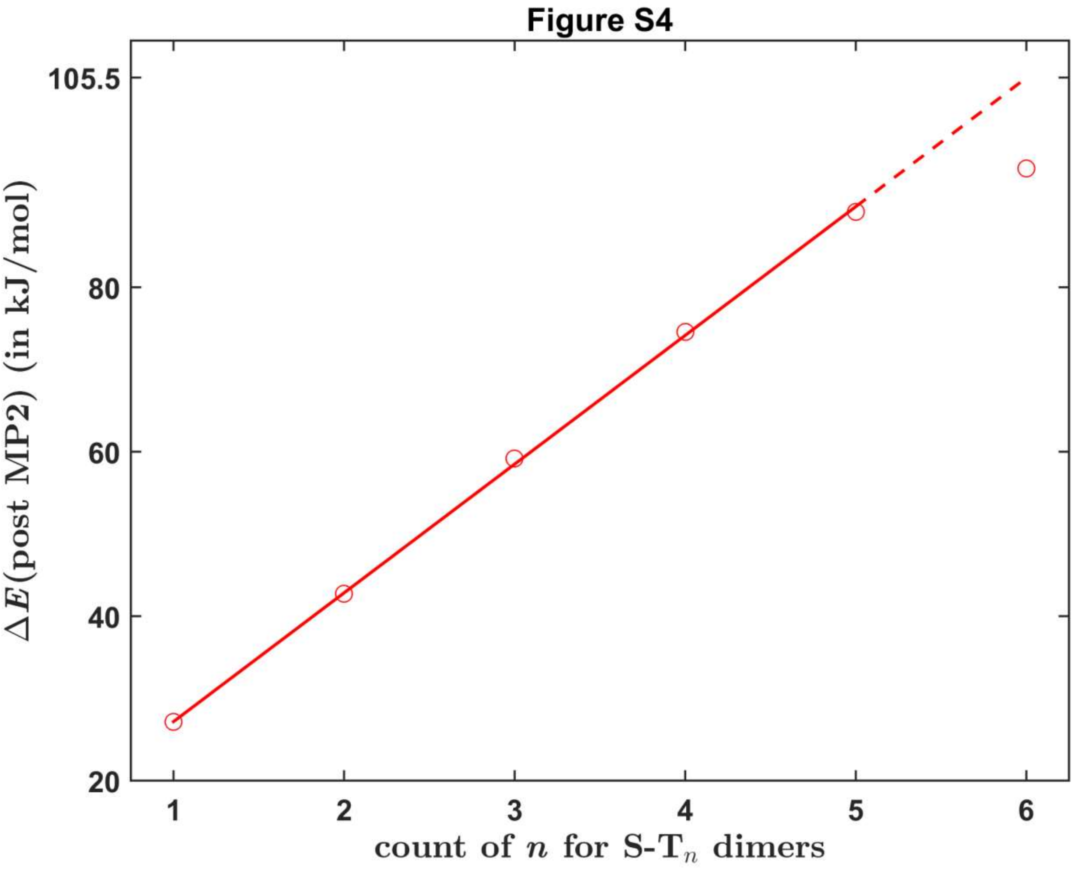

Figure S5. The dependence of the B97-D/def2-TZVPP interaction energy (denoted as $\Delta E(\mathrm{DFT})$) upon molecular size of investigated dimers. The red line is $\{\Delta E(\mathrm{DFT})\} = a \times \{n-1\} - 40.961$ kJ/mol with $a = -16.27$ that has the standard deviation of 0.35.

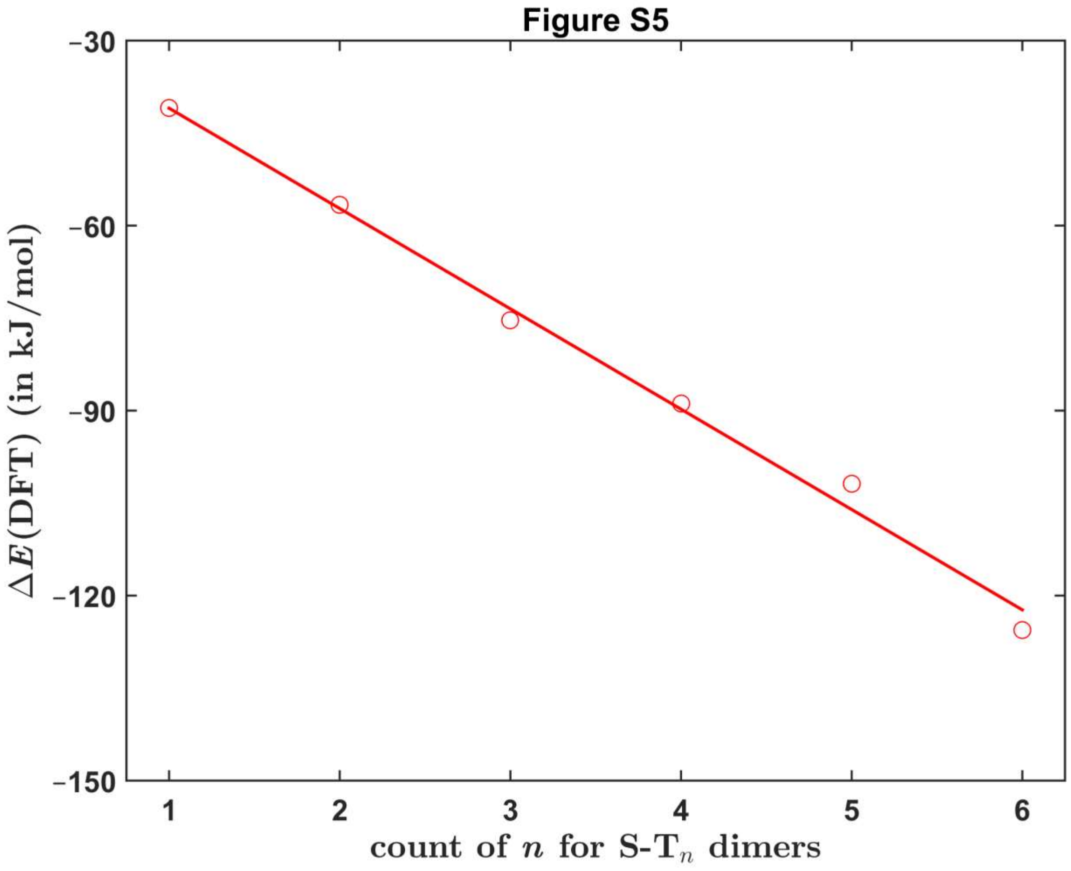

Figure S6. The chain of atoms defining the dihedral angle β in **S-T**$_1$ dimers investigated in main text.

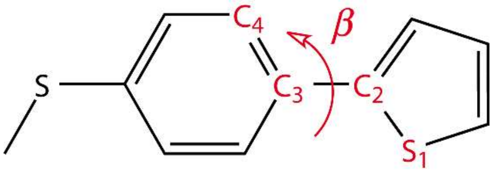

Figure S7. The chain of atoms defining the dihedral angle β in **S-T**$_2$ dimers investigated in main text.

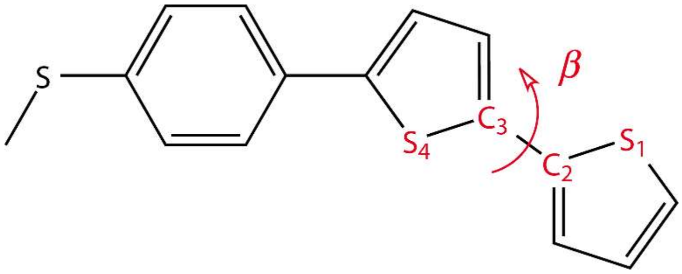

Supplement: Supplementary file 1 [file ijms-24-13349-s001.zip › SI.pdf]
